# Association between abnormal fetal head growth and autism spectrum disorder

**DOI:** 10.1192/j.eurpsy.2021.364

**Published:** 2021-08-13

**Authors:** O. Regev, G. Cohen, A. Hadar, G. Meiri, H. Flusser, A. Michaelovski, I. Dinstein, R. Hershkovitz, I. Menashe

**Affiliations:** 1 Department Of Public Health, Ben-Gurion University of the Negev, Beer Sheva, Israel; 2 Department Of Obsetrics And Gynecology, Clalit Health Services, Beer Sheva, Israel; 3 Child Psychiatry, Soroka Unversity Medical Center, Beer Sheva, Israel; 4 Child Development Center, Soroka University Medical Center, Beer Sheva, Israel; 5 Department Of Psychology, Ben-Gurion University of the Negev, Beer Sheva, Israel; 6 Department Of Obsetrics And Gynecology, Soroka University Medical Center, Beer Sheva, Israel

**Keywords:** autism spectrum disorder, Prenatal Ultrasound, Brain Development

## Abstract

**Introduction:**

Despite evidence for the prenatal onset of abnormal head growth in ASD children, studies on fetal ultrasound data in ASD are limited and controversial.

**Objectives:**

To understand whether people with ASD have abnormal head growth during gestation

**Methods:**

A longitudinal matched case-sibling-control study on prenatal ultrasound biometric measures of ASD children was conducted. Children with ASD were matched to two control groups: (1) typically developed sibling (TDS) and (2) typically developed population (TDP). The cohort comprised 528 children (72.7% males): 174 ASD, 178 TDS, and 176 TDP.

**Results:**

Second-trimester ASD and TDS fetuses had significantly smaller biparietal diameter (BPD) than TDP fetuses (aOR_zBPD_=0.685, 95%CI=0.527-0.890 and aOR_zBPD_=0.587, 95%CI=0.459-0.751, respectively). However, these differences became statistically indistinguishable in the third trimester. Head biometric measures were associated with the sex of the fetus, with males having larger heads than females within and across groups. A linear mixed-effect model assessing the effects of sex and group assignment on fetal longitudinal head growth indicated faster BPD growth in TDS vs both ASD and TDP in males (β=0.084 and β=0.100 respectively; p<0.001) but not in females, suggesting an ASD–sex interaction in head growth during gestation. Fetal head shape showed sex-specific characteristics, and head growth was inversely correlated with ASD severity in males and females, thus further supporting the sex effect on the association between fetal head growth and ASD.

**Conclusions:**

Our findings suggest that abnormal fetal head growth is a familial trait of ASD, which is modulated by sex and is associated with the severity of the disorder.

**Disclosure:**

No significant relationships.

